# Commentary: Are geographical “cold spots” of male circumcision driving differential HIV dynamics in Tanzania?

**DOI:** 10.3389/fpubh.2016.00046

**Published:** 2016-03-30

**Authors:** Ereny Ibrahim, Shabnam Asghari

**Affiliations:** ^1^Primary Healthcare Research Unit, Faculty of Medicine, Memorial University, St. John’s, NL, Canada

**Keywords:** ecological fallacy, ecological studies, medical geography, cold spot analysis, HIV, male circumcision

Availability of geographic information systems and the expansion of methodological approaches in geospatial analysis over the past decade have made studies focusing on the spatial distributions of disease increasingly more common (1). These types of investigations are valuable in identifying locations with unexpectedly high or low rates of disease. They also allow for the testing of hypotheses that look to find relationships between the prevalence of a disease and environmental factors. In practice, these studies are known as ecological studies and are often conducted using secondary data. They are efficient and cost-effective and allow for the exploration and generation of hypotheses; however, they are not able to provide a strong cause-and-effect relationship between a set of variables.

It is often suggested that the findings from ecological studies require careful interpretation and consideration of their limitations such as ecological fallacy (2). Cuadros et al. recently suggested a geographic association between male circumcision (MC) and HIV in Tanzania using data from demographic and health surveys (TDHS) (3). The authors also suggest MC as being a factor associated with the distribution of HIV in Tanzania. They concluded that MC was an influential factor in HIV prevalence among men and women in Tanzania.

Before accepting the authors’ comments, we would like to point out the importance of considering ecological fallacy when using health data in geospatial analysis. Ecological fallacy is a logical fallacy that occurs when a conclusion about individuals are made based on the analysis of group-level data (4). A relationship between exposure and disease frequency at the group level in an ecological study does not infer that such a relationship exists at the individual level (5). According to Willian Robinson, there is a statistical difference between the correlation of variables aggregated by group or geographic area and correlation established on individual-level variables of that same population (6).

In this article, HIV testing was completed for all tested men and women; subsequently, the prevalence of HIV and the prevalence of MC were assessed at each sample location. Estimates of HIV prevalence and MC were based on aggregated data for specified geographic units. As a result, there is confusion between aggregate and individual inference. The authors used the aggregated data for prevalence of MC in a given area to detect the effect it had on male HIV risk. They concluded that there is a significant spatial association between MC and HIV. In areas with a low percentage of circumcised males, a large proportion of the HIV epidemic is concentrated. This spatial correlation between MC and HIV does not necessarily mean that there is a correlation at the individual level. This study gave up the ability to perform individual-level data analysis by performing aggregate analyses, which provide a picture of group-level outcomes (cities).

There are different components of ecological fallacy that can be found in this article:

- Ecological correlation: correlation between variables (HIV and MC) is on group level, in contrast to a correlation between these variables that describe individuals.
- Ecological rates: HIV rates in men and women were estimated *via* geographic units, in contrast to the rates that describe total rates on individual level.
- Confusion between higher rates and higher likelihood; where the units of analysis are geographic units, higher MC rates versus the likelihood of being HIV positive at the group level does not imply causation.

On the other hand, the authors subsequently used the same variable and applied it to females, assuming the reduced risk for infection was transferrable to the opposite sex, since they live within the same area as a given set of circumcised males. Even with the assumption of high-quality data and accurate sampling strategy, the drop in HIV prevalence for circumcised males for a given area does not necessarily equate to an increased or decreased risk of infection for females living in the same area.

In conclusion, this study generates a hypothesis and suggests a possible association between MC and HIV; however, the findings of this study should be interpreted in light of its limitations as an ecological study. Further research on individual-level data with a stronger study design is a requirement to support a cause-and-effect relationship between MC and HIV. It is necessary to provide a model using individual-level data and then model how the individual and group levels are associated, and finally assess whether the group-level model will add to the understanding of this association.

## Author Contributions

EI wrote the first draft of the manuscript. SA reviewed the manuscript and provided comments.

## Conflict of Interest Statement

The authors declare that the research was conducted in the absence of any commercial or financial relationships that could be construed as a potential conflict of interest.
